# Mohs Surgery Price Transparency and Variability at Academic Hospitals After the Implementation of the Federal Price Transparency Final Rule

**DOI:** 10.2196/50381

**Published:** 2023-11-15

**Authors:** Neelesh P Jain, Christian Gronbeck, Eric Beltrami, Hao Feng

**Affiliations:** 1 Department of Dermatology University of Connecticut Farmington, CT United States; 2 School of Medicine University of Connecticut Farmington, CT United States

**Keywords:** Mohs micrographic surgery, Mohs surgery, Mohs, dermatologic surgery, price transparency, healthcare costs, health care costs, healthcare policy, health care policy, dermatology, dermatological, surgery, surgical, Medicare, insurance, coverage, cost, costs, economic, economics, fee, fees, price, prices, pricing, transparency, reporting

## Introduction

In response to rising health care costs, which can lead to high out-of-pocket patient costs, the US federal government implemented the Hospital Price Transparency Final Rule in 2021 [1,2]. This legislation mandates that hospitals disclose cash and commercial insurance prices for at least 300 medical services. The goal was to foster price transparency, stimulate price competition, and ultimately lower health care costs. As use of Mohs micrographic surgery (MMS) continues to expand, understanding the cost variability of this procedure across hospitals and geographic regions is crucial. Our study aimed to elucidate the current landscape of price transparency and variability for MMS procedure costs at academic hospitals, inclusive of facility and physician fees.

## Methods

### Overview

To ensure the hospitals evaluated offered MMS, we limited our selection criteria to academic hospitals with MMS fellowships. Private clinics were excluded as they are not subject to the Price Transparency Rule. Using Turquoise Health, a company that compiles nationwide price information from hospitals, we evaluated hospital-reported cash and commercial insurance prices for Current Procedural Terminology (CPT) code 17311 for the calendar year 2022; additional MMS CPT codes (17312-17315) were not reported by hospitals [3]. For reference, we gathered Medicare-reported facility and physician fees, adjusted by state [4]. We calculated the percentage of hospitals reporting cash and commercial insurance prices and compared median prices by payment type.

### Ethical Considerations

This study used publicly available online data sets and did not qualify as human subject research; therefore, institutional review board approval was not required at the University of Connecticut Health Center.

## Results

Among 62 hospitals, 36 (58.1%) reported commercial insurance prices and 27 (43.5%) reported cash prices, with 26 (41.9%) reporting both. Hospitals in the Northeast more frequently reported cash prices as compared to other regions (73.7% vs 27.3%-35.7%, *P*=.02); regional differences in commercial insurance price reporting did not reach significance (*P*=.16). Hospitals in the Northeast reported the highest median cash prices ($1266.8 vs $514.8-$838.7, *P*=.04); regional differences in median commercial insurance prices did not reach significance (*P*=.07). Across all hospitals, cash prices were more frequently (n=16, 59.3%) higher than commercial prices (Table 1).

**Table 1:** Price^a^ reporting and variation for Mohs micrographic surgery by payor type among academic hospitals.

Region and payor type		Hospitals reporting price, n (%)	Price (US $), median (IQR)	Payor type as lowest reported price, n (%)^b^
**All regions (N=62 hospitals)**				
	Cash	27 (43.5)	838.7 (585.6-1711.8)	16 (59.3)
	Commercial	36 (58.1)	717.4 (539.2-1330.0)	11 (40.7)
	Medicare, facility fee	—^c^	457.9 (432.7-527.3)	—
	Medicare, facility plus physician fees	—	806.1 (780.2-886.2)	—
**Northeast (n=19 hospitals)**				
	Cash	14 (73.7)	1266.8 (690.4-1856.2)	11 (78.6)
	Commercial	15 (78.9)	707.3 (633-1135.8)	3 (21.4)
	Medicare, facility fee	—	459.6 (457.9-574.6)	—
	Medicare, facility plus physician fees	—	819.2 (805.6-971.2)	—
**Midwest (n=14 hospitals)**				
	Cash	5 (64.3)	514.8 (494.0-585.6)	3 (60)
	Commercial	7 (50)	531.8 (513.0-539.9)	2 (40)
	Medicare, facility fee	—	441.7 (432.7-459.7)	—
	Medicare, facility plus physician fees	—	786.7 (765.4-808.1)	—
**South (n=18 hospitals)**				
	Cash	5 (27.8)	773.0 (461.5-827.1)	1 (25)
	Commercial	8 (55.6)	1254.5 (700.9-1831.7)	3 (75)
	Medicare, facility fee	—	429.6 (403.9-437.2)	—
	Medicare, facility plus physician fees	—	780.3 (767.2-790.7)	—
**West (n=11 hospitals)**				
	Cash	3 (27.3)	838.7 (542.3-1214.9)	0 (0)
	Commercial	6 (54.5)	1178.2 (686-1330)	3 (100)
	Medicare, facility fee	—	681.6 (527.3-681.6)	—
	Medicare, facility plus physician fees	—	1058.8 (886.2-1058.8)	—

^a^Hospital-reported median cash prices, commercial insurance prices, and reference Medicare facility and physician fees for Mohs micrographic surgery. Commercial insurance prices for each hospital indicate the median across all payors (eg, UnitedHealth, Anthem, Humana, etc) as reported by the hospital. While the intention of the Hospital Price Transparency Final Rule is to provide comparable holistic pricing information, certain hospitals include only hospital facility fees while others additionally include physician fees in the reported prices. For this reason, Medicare facility and physician fees are provided for contextual purposes but direct comparisons to the hospital-reported prices are not made.

^b^Analysis only conducted for hospitals with both prices listed; at 1 hospital, median cash and commercial prices were equivalent.

^c^Not applicable.

## Discussion

### Principal Findings

The findings indicate that fewer than half of hospitals reported both cash and commercial insurance prices for MMS, and median prices varied substantially across payor types and regions. This is consistent with findings in other surgical fields [1,5-7]. Regional variations may be partially explained by studies that have shown a hospital’s compliance with the Price Transparency Rule is most strongly associated with the compliance status of its peer hospitals in the same area [8]. Interestingly, cash prices tended to be the highest, possibly because this helps hospitals offset losses incurred treating uninsured patients. Other studies have shown that compliance with the Price Transparency Rule is below 30%, yet only 2 hospitals have been fined for noncompliance [1]. The cost of compliance, requiring adequate information technology expertise and personnel, can be a barrier to hospitals with fewer financial resources [8]. Strategies to increase compliance include implementing positive incentives, proper enforcement, and increased financial penalties [9]. Many MMS procedures are performed in private clinics, which the Price Transparency Rule does not apply to. Fully enabling price shopping for MMS would require the Price Transparency Rule mandating MMS prices be reported by both hospital systems and private clinics. Additionally, pricing information would need to be easier for patients to access, comprehend, and compare.

### Conclusion

Our findings indicate that significant variability and opacity exist in MMS pricing at academic hospitals. Across all of health care, pricing is not often clearly defined or publicly available. This ambiguity can be confusing for both health care providers and patients, possibly leading to wider cost variability and hindered health care access for select patients. Additional studies exploring health care costs may help shed light on the factors influencing price variability. Limitations to this study include the inability to generalize to nonacademic hospital settings such as private clinics, which perform many MMS procedures but to which the Price Transparency Rule does not apply. Additionally, benchmarking to Medicare pricing, which contains well-delineated facility and physician fees, is difficult as not all hospitals report both fee components despite the intention of the Price Transparency Rule to provide a complete picture of the total cost for a given service [2]. Nonetheless, this analysis provides an important initial characterization of the current state of MMS pricing transparency and variability at academic hospitals.

## Abbreviations

CPT: Current Procedural Terminology
MMS: Mohs micrographic surgery
